# Effects of Recurring IPC vs. rIPC Maneuvers on Exercise Performance, Pulse Wave Velocity, and Red Blood Cell Deformability: Special Consideration of Reflow Varieties

**DOI:** 10.3390/biology11020163

**Published:** 2022-01-20

**Authors:** Marijke Grau, Benedikt Seeger, Lukas Mozigemba, Roland Roth, Luca Baumgartner, Hans-Georg Predel, Wilhelm Bloch, Fabian Tomschi

**Affiliations:** 1 Institute of Cardiovascular Research and Sports Medicine, Department of Molecular and Cellular Sports Medicine, German Sport University Cologne, Am Sportpark Müngersdorf 6, 50933 Cologne, Germany; benediktseeger@web.de (B.S.); l.mozigemba@web.de (L.M.); ron91@gmx.net (R.R.); lucaba@gmx.de (L.B.); w.bloch@dshs-koeln.de (W.B.); tomschi@uni-wuppertal.de (F.T.); 2 Institute of Cardiovascular Research and Sports Medicine, Department of Preventive and Rehabilitative Sports and Performance Medicine, German Sport University Cologne, Am Sportpark Müngersdorf 6, 50933 Cologne, Germany; predel@dshs-koeln.de; 3 Department of Sports Medicine, University of Wuppertal, Moritzstraße 14, 42117 Wuppertal, Germany

**Keywords:** remote ischemic preconditioning, ischemic preconditioning, blood reflow, performance, red blood cell deformability, pulse wave velocity, blood pressure

## Abstract

**Simple Summary:**

Ischemia preconditioning (IPC) and remote (r) IPC consist of a sequence of blood occlusion and blood reflow to manipulate an effector organ either near (IPC) or remote (rIPC) from the occlusion site. Its benefits in medical applications are well-documented, while there is no consensus regarding its effects on exercise performance, arterial stiffness, blood pressure, and red blood cell deformability. The aim of the study was to test and compare the effects of five daily recurring IPC and rIPC maneuvers on these parameters. Additionally, different reflow protocols, low (LR) and high reflow (HR), were compared to investigate potential differences of the shear stress occurring during reflow on the outcome. Results of thirty male participants suggest improved exercise performance during IPC LR and to a lesser extent rIPC LR interventions, while HR conditions did not affect performance parameters. This might in part be related to increased red blood cell deformability and reduced lactate production, while relations to altered pulse wave velocity or blood pressures seem less likely. In conclusion, the site of occlusion and reflow conditions might influence the effectiveness of the (r)IPC intervention, which should be considered in sport-specific but also clinical settings.

**Abstract:**

Beneficial effects of (remote) ischemia preconditioning ((r)IPC), short episodes of blood occlusion and reperfusion, are well-characterized, but there is no consensus regarding the effectiveness of (r)IPC on exercise performance. Additionally, direct comparisons of IPC and rIPC but also differences between reflow modes, low reflow (LR) and high reflow (HR) in particular, are lacking, which were thus the aims of this study. Thirty healthy males conducted a performance test before and after five consecutive days with either IPC or rIPC maneuvers (*n* = 15 per group). This procedure was repeated after a two-week wash-out phase to test for both reflow conditions in random order. Results revealed improved exercise parameters in the IPC LR and to a lesser extent in the rIPC LR intervention. RBC deformability increased during both rIPC LR and IPC LR, respectively. Pulse wave velocity (PWV) and blood pressures remained unaltered. In general, deformability and PWV positively correlated with performance parameters. In conclusion, occlusion of small areas seems insufficient to affect large remote muscle groups. The reflow condition might influence the effectiveness of the (r)IPC intervention, which might in part explain the inconsistent findings of previous investigations. Future studies should now focus on the underlying mechanisms to explain this finding.

## 1. Introduction

Ischemia preconditioning (IPC) and remote IPC (rIPC) were developed to protect specific organs, such as the heart, against ischemia/reperfusion (I/R) injury. Thereby, formation of reactive oxygen species (ROS) and oxidative stress [1] are known mediators of I/R injury. During (r)IPC, short episodes of blood occlusion/ischemia and blood reperfusion (I/R) are applied to areas either remote from the effector organ (rIPC) or nearby (IPC). One of the first non-invasive protocols applied comprised four consecutive cycles of five minutes of ischemia followed by five minutes of reperfusion. Blood occlusion is achieved by inflation of a blood pressure cuff to a final pressure above the systolic blood pressure to limit the blood flow [2]. Observed protective effects of (r)IPC involved the release of multiple signaling molecules and mechanisms, including the upregulation of oxidative stress defense genes [3] and nitric oxide (NO) generation [4]. NO is enzymatically produced by NO synthases (NOS) [5,6,7] and represents a molecule known to regulate endothelium-dependent blood flow, vascular resistance, aortic and systemic arterial stiffness [8], and blood pressure, but is also involved in red blood cell (RBC) deformability regulation [9]. Remote ischemia preconditioning was described to also increase RBC-NOS activation, concomitant NO levels, and finally, RBC deformability [10], suggesting diverse effects of NO during (r)IPC. The deformability of RBC is defined as the ability of the cells to change their shape at a given shear stress and is of crucial importance for the oxygen supply of the body cells within the microcirculation. Situations related to an increased oxygen demand, such as physical exercise, might thus be associated with higher deformability values of the RBC. Indeed, studies indicate a positive relation between training, RBC function, and exercise performance [11,12,13,14]. Given the various positive effects of (r)IPC observed in a clinical setting, this technique has been widely applied in sports. However, recent reviews revealed that only few studies describe a positive relation between (r)IPC application and exercise performance [15,16,17,18]. Most investigations that aim to depict performance-enhancing effects of IPC conducted IPC protocols on the legs, meaning that the blood flow of the legs was occluded, and the legs were engaged in the subsequent performance test, e.g., bicycle ergometer or treadmill running. However, IPC was also conducted at a remote body part, such as the arms, that is not primarily engaged in the ergometer exercise. This type of IPC is then referred to as remote ischemic preconditioning (rIPC) [19]. rIPC might be better tolerated by the study participants since they reported a discomfort when the occlusion site was the same as the executive muscle group. Positive effects of (r)IPC on exercise performance were related to a promoted phosphocreatine resynthesis, improved functioning of the mitochondrial ATP-dependent potassium channels, attenuated ATP depletion, enhanced metabolic efficiency, and increased vasodilation by the above-mentioned NO-dependent pathways. Thus, oxygen delivery and extraction were enhanced, and subsequent physical performance was ameliorated [20,21]. The remaining heterogeneity of the results might be explained by the differences in the reported study settings, including variations in the number of applied I/R cycles, the applied pressure within the blood pressure cuff, the application site (arm(s) or leg(s) or both), the study cohort, and the associated performance test [16,17,18]. These analyzed studies only tested the effects of a single I/R cycle on exercise performance and thus, possible cumulative effects of (r)IPC are less described. Furthermore, direct comparisons of rIPC to IPC in order to assess the extent of effectiveness of these two interventions are lacking. Finally, the reperfusion phase described in all studies was characterized by an immediate release of pressure from the blood pressure cuff, which results in a prompt filling of the blood vessels. Whether the high shear forces that result from this procedure might attenuate the potential benefits of (r)IPC during the following exercise remains unknown and requires a direct comparison of the prompt to a moderated filling phase.

Thus, the primary objective of this study was to evaluate the different effects of recurrent rIPC maneuvers compared to a conventional IPC intervention on performance. In parallel, different reflow protocols, conventional high reflow, and now the implemented low reflow conditions were compared. Secondary objectives were to gauge whether these interventions alter arterial stiffness, blood pressure, and RBC deformability and whether these changes correlate to the tested exercise performance.

## 2. Materials and Methods

### 2.1. Study Participants

A total of *n* = 30 healthy male volunteers participated in this study. Participants were randomly assigned to one of the two study groups with *n* = 15 each. Anthropometric parameters of the study participants were assessed upon first arrival at the laboratory using the calibrated Seca^®^ medical Body Composition Analyzer (mBCA 515, Seca GmbH & Co.KG., Hamburg, Germany). Data of the two study groups were (age (years), height (cm), weight (kg)): Group 1 rIPC: 25.6 ± 3.4, 182.3 ± 5.0, 80.7 ± 6.2, and Group 2 IPC: 25.9 ± 3.2, 182.7 ± 5.6, 82.3 ± 7.8. Participants were non-endurance athletes and reported an average of 3–4 training sessions per week with an average of 60 min duration per training session, which was assessed via questionnaires. Training duration did not significantly differ between the study groups. Exclusion criteria included smoking, metabolic or cardiovascular disease, regular use of medication or dietary supplements, and blood donation within the last three months because this might impact the study outcomes. Participants’ exercise routines were retained during the study, but rigorous training and consumption of alcohol was prohibited 24 h prior to the exercise testes. The protocols applied here were in accordance with the Declaration of Helsinki and the study was approved by the ethics committee of the German Sport University Cologne (# 052/2018–9 May 2018). All participants were informed about the procedure and possible risks and signed an informed consent document prior to investigations.

### 2.2. Study Design: General Information

**Figure 1:**
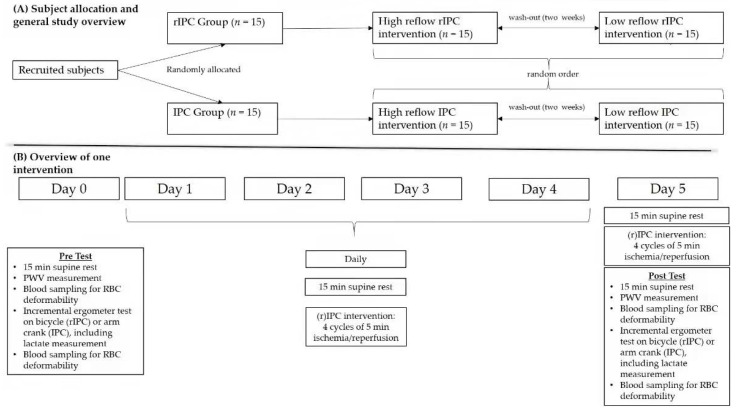
Schematic representation of the study design. (**A**) Allocation of participants and general study overview and (**B**) overview of one intervention.

To test and compare the effects of rIPC to IPC, the study population was separated into two groups. Group1—rIPC: A blood pressure cuff (Rudolf Riester Riester GmbH, Jungingen, Germany) was placed on the left upper arm and pressure was set to 200 mmHg, thus restricting the blood flow. The performance test was conducted using a bicycle ergometer. Group 2—IPC: Placing of and pressure within the blood pressure cuff were comparable to the rIPC group. The performance test was conducted using an arm crank ergometer. The study protocol of both groups was comparable in length, frequency of the applied ischemia and reperfusion cycles, and the scheduled pre- and post-exercise tests (for details, see below). All study groups performed two different reperfusion protocols: low reflow (LR) reperfusion was defined as the uniform release of the pressure from the blood pressure cuff with 33 mmHg released per 10 s, resulting in a complete deflation of the cuff within one minute, while the high reflow (HR) reperfusion describes the sudden release of the pressure. Thus, four different conditions were conducted: rIPC low reflow/high reflow and IPC low reflow/high reflow. The sequence of the reflow protocols varied between the subjects and a wash-out phase of at least two weeks was scheduled between the different reflow protocols to prevent carry-over effects [4,22] (Figure 1).

### 2.3. Study Protocol: Detailed Demonstration

#### 2.3.1. Blood Pressure and Pulse Wave Velocity Measurement

Upon arrival, the participants were asked to rest for 15 min in supine position without moving. A blood pressure cuff was placed on their left upper arm. Then, pulse wave velocity and central as well as peripheral blood pressure (BP) were measured using the Mobil-O-Graph (IEM GmbH, Aachen, Germany), an automated oscillometric arm cuff-based ambulatory blood pressure monitoring device. First, the device measures the peripheral BP. After 30 s of pause, the device automatically measures additional hemodynamic parameters including pulse wave velocity (PWV) and central BP, which are presented in the software output. The time duration of the entire measurement was three to four minutes.

#### 2.3.2. Initial Exercise Test, T0

Following these measurements, all participants performed an incremental exercise test either on a bicycle ergometer (rIPC group) (Ergoline Ergometrics 800, Ergoline GmbH, Bitz, Germany) or an arm crank ergometer (IPC group) (Ergoline Ergometer Ergoselect 400, Ergoline GmbH, Bitz, Germany). Thus, because the maximal workload for arm cranking is less than 50% compared to cycling, the initial work load and work load increments for the IPC group were reduced by 50% compared to the rIPC group [23]. All exercise interventions were supervised by the same trained sport scientists.

First, 2 × 20 µL of blood was sampled from the earlobe to measure lactate concentration and RBC deformability, respectively. The performance test started with an initial work load of 50 watt (rIPC)/25 watt (IPC) with an increase of 50/25 watt every two minutes and a cadence of 60–80 revolutions per minute (rpm). Tests were terminated at subjective exhaustion or when the rpm were below 60 rpm for more than 30 s. After each increment and at the end of the test, additional capillary blood was sampled from the earlobe for lactate analysis. Another 20 µL of capillary blood was sampled for the RBC deformability measurement. Finally, time to exhaustion (TTE) and power at exhaustion were recorded.

#### 2.3.3. Ischemia (Blood Occlusion)/Reperfusion Maneuver

The blood occlusion/reperfusion maneuvers were similar for the rIPC and IPC groups and were carried out on the following five consecutive days with one maneuver per day. The examinations took place at the same time of day to prevent diurnal changes. Before every maneuver, each subject was asked to rest in supine position for 15 min. This measure intended to adapt to postural changes from standing to lying position.

A conventional blood pressure cuff was placed on the left upper arm and was inflated to 200 mmHg to limit the blood flow to the forearm and hand. A pulse oximeter (ri-fox n pulse oximeter, Rudolf Riester Riester GmbH, Jungingen, Germany) was placed on the left index finger to monitor and verify the absence of a measurable pulse. The duration of this occlusion phase was 5 min, followed by the reperfusion phase (either high reflow or low reflow). The next occlusion phase started 5 min after the end of the previous reperfusion phase. A total of four occlusion/reperfusion cycles were applied, respectively. Before and after the last occlusion/reperfusion maneuver on day five, another 20 µL of capillary blood was taken to measure RBC deformability.

#### 2.3.4. Final Exercise Test, T1

A post-exercise test, comparable to the pre-test described above, was performed after the last (r)IPC maneuver with the same parameters recorded as during the initial exercise test (see Figure 1).

### 2.4. Sample Measurement

#### 2.4.1. Lactate Analysis

Capillary blood was mixed with 1 mL of hemolytic liquid using prepared tubes from EKF-diagnostics GmbH (Barleben, Germany). Lactate levels of the samples were analyzed using an EKF-Biosen S-Line Lab + lactate analyzer (EKF-diagnostics GmbH, Barleben, Germany). Two and four mmol/L lactate thresholds were calculated, and the respective watt power and time at the two and four mmol/L lactate threshold were calculated using a calculation matrix developed at the German Sport University, as previously described [24].

#### 2.4.2. RBC Deformability Measurement

Capillary blood was mixed with 5 mL of polyvinylpyrrolidone solution (PVP, viscosity 31.24 cP) to measure RBC deformability using the laser-assisted optical rotational cell analyzer (LORCA, RR Mechatronics, Zwaag, The Netherlands) [25]. The LORCA system applied different fluid shear stresses to alter the RBC deformability, which was monitored by laser diffraction analysis. More precisely, the sampled blood/PVP samples were sheared in a glass Couette system with a laser beam directed through the blood/PVP sample. The resulting laser diffraction pattern influenced by the deformation status of the RBC was analyzed by the LORCA software and resulted in an elongation index (EI). The EI was calculated for nine consecutive shear stresses between 0.3 and 50 Pa [26]. For comparisons of RBC deformability values, maximum deformability at infinite shear stress (EImax) and shear stress at one-half EImax (SS 1/2) were provided by the LORCA software, and the SS1/2:EImax ratio was calculated [27], with the latter being presented herein. A lower SS1/2:EImax ratio indicates higher RBC deformability.

### 2.5. Statistics

Statistical analyses of the data were performed using GraphPad Prism 8.0.2 (GraphPad Software, San Diego, CA/USA). Data are presented as mean ± standard deviation (SD). The Shapiro–Wilk test indicated a normal (Gaussian) distribution of the data. For rIPC and IPC, differences of the protocols in TTE, PWV, and maximum lactate concentration were assessed by a two-way mixed analysis of variance (ANOVA) with the factors ‘protocol’ (high reflow, low reflow) and ‘time point’ (pre, post). Furthermore, to detect differences in RBC deformability, the effects of the protocol × time point interaction were calculated. If differences were observed, Tukey post hoc analyses were conducted to determine simple main effects. An overall type I error rate of 0.05 was used as an indication of statistical significance for each calculation. Delta (Δ) values were calculated by subdividing the pre-value from the respective post-value. This was carried out for PWV, RBC deformability, and central systolic and diastolic blood pressure, as well as for time to exhaustion. Pearson correlation analyses using the calculated Δ values were performed to further test the effect of PWV, RBC deformability, and central blood pressure on performance outcome, and respective r-values > 0.5 were provided when appropriate.

## 3. Results

### 3.1. Performance Parameters

**Figure 2:**
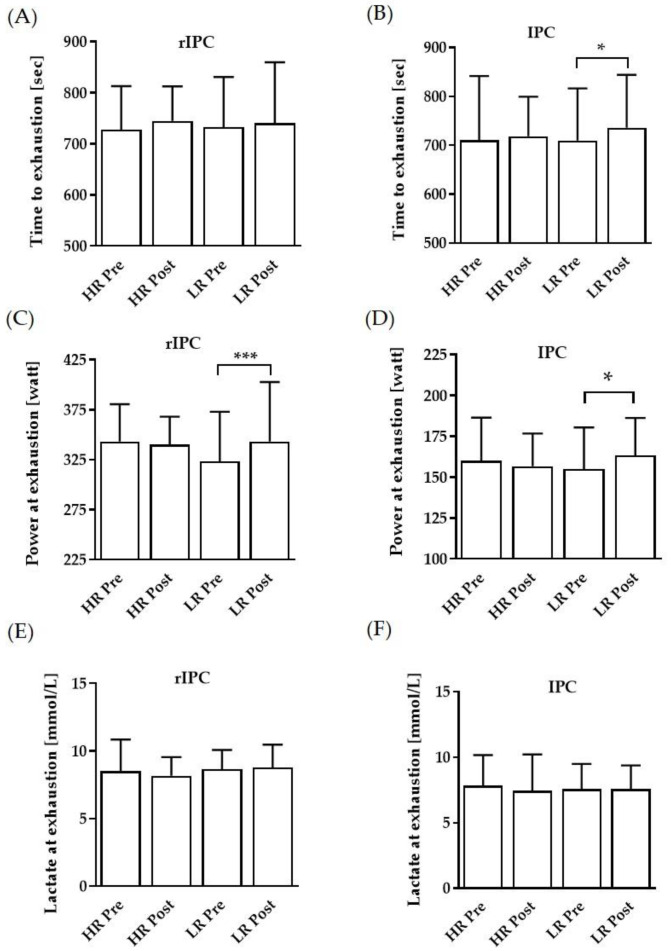
Pre- and post-performance data of the respective interventions. Time to exhaustion before and after the low reflow (LR) and high reflow (HR) (**A**) rIPC intervention and (**B**) IPC intervention, respectively. Power at exhaustion before and after the LR and HR (**C**) rIPC and (**D**) IPC intervention, respectively. Lactate at exhaustion after the LR and HR (**E**) rIPC intervention and (**F**) IPC intervention, respectively. Data are shown as mean (SD); * *p* < 0.05 and *** *p* < 0.001.

TTE was significantly prolonged in the IPC low reflow protocol, while no such change was observed in the rIPC protocols (Figure 2A,B). Power at exhaustion was significantly higher in both rIPC and IPC low reflow protocols (Figure 2C,D). Lactate concentrations at exhaustion were comparable between the groups (Figure 2E,F).

### 3.2. Performance at 2 and 4 mmol/L Thresholds

**Figure 3:**
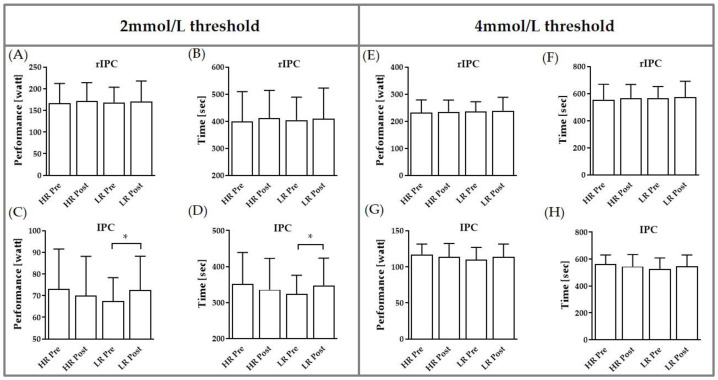
Pre- and post-performance data at the 2 and 4 mmol/L lactate thresholds of the respective interventions. (**A**) Performance (watt) and (**B**) time (seconds) at the 2 mmol/L threshold during the low reflow (LR) and high reflow (HR) rIPC interventions, respectively. (**C**) Performance (watt) and (**D**) time (seconds) at the 2 mmol/L threshold during the LR and HR IPC interventions, respectively. (**E**) Performance (watt) and (**F**) time (seconds) at the 4 mmol/L threshold during the low reflow (LR) and high reflow (HR) rIPC interventions, respectively. (**G**) Performance (watt) and (**H**) time (seconds) at the 4 mmol/L threshold during the LR and HR IPC interventions, respectively. Data are shown as mean (SD); * *p* < 0.05.

In the rIPC protocol, both performance and time at 2 and 4 mmol/L remained unaltered (Figure 3A,B,E,F). In the IPC protocol, performance and time at the 2 mmol/L threshold were significantly increased in the post-test, but only in the low reflow protocol (Figure 3C,D). In contrast, performance and time at the 4 mmol/L threshold were comparable between the test conditions (Figure 3G,H).

### 3.3. Pulse Wave Velocity and Blood Pressures

**Figure 4:**
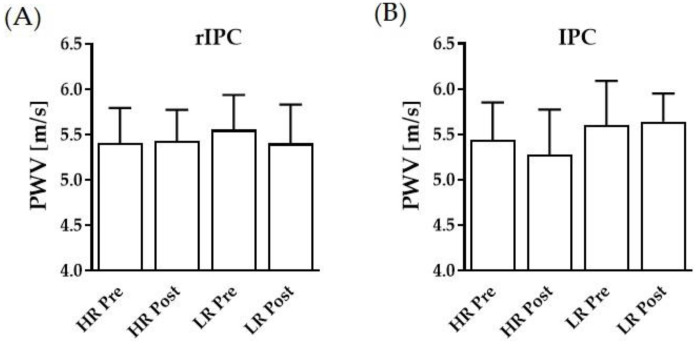
Pre- and post-intervention values of pulse wave velocity. Pulse wave velocity before and after the (**A**) low reflow (LR) and high reflow (HR) rIPC and (**B**) IPC interventions, respectively. Data are shown as mean (SD).

Data suggest no effect of the tested rIPC and IPC protocols nor of the reperfusion modes (HR, LR) on pulse wave velocity (Figure 4A,B). However, correlation analysis of PWV and TTE revealed a moderate negative correlation (r = –0.6505; *p* = 0.0086), showing that lower PWV was associated with prolonged TTE. Both central and peripheral systolic and diastolic blood pressures remained unaltered during the interventions and between the reflow conditions (Table 1).

### 3.4. RBC Deformability

**Figure 5:**
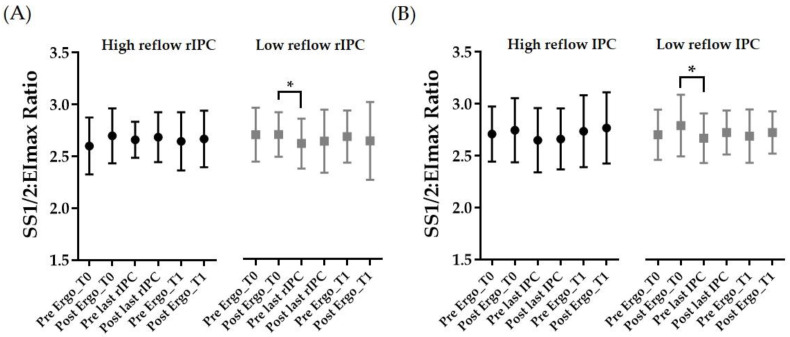
Red blood cell deformability during the (r)IPC intervention and performance test. Red blood cell deformability, represented as the SS1/2:EImax ratio, during (**A**) high reflow (HR) rIPC (left panel) and low reflow (LR) rIPC (right panel) interventions, and during (**B**) HR IPC (left panel) and LR IPC (right panel) interventions, respectively. Data are shown as mean (SD); * *p* < 0.05.

SS1/2:EImax values and thus RBC deformability remained unaffected during HR rIPC and HR IPC, respectively (Figure 5A,B, left panels, respectively). During LR rIPC and LR IPC, SS1/2:EImax values decreased, reflecting increased deformability, from post ergo T0 (pretest) to prior to the last (r)IPC intervention (*p* < 0.05). The SS1/2:EImax ratio decreased between these two time points by –3.53% during LR rIPC and by –3.73% during LR IPC (Figure 5A,B). The correlation between the SS1/2:EImax ratio and TTE was negative (*r* = –0.6091; *p* = 0.0271), indicating that higher RBC deformability is associated with prolonged TTE.

## 4. Discussion

Episodes of ischemia and blood reperfusion were suggested to positively influence exercise performance. Both stimuli near (IPC) and remote (rIPC) from the effector region were reported to affect performance parameters [18]. However, direct comparisons of rIPC and IPC but also investigations of different reflow intensities were lacking and needed investigation due to inconsistent reports in the literature. Finally, mechanistic explanations of possible performance changes should be provided. The main finding of the present study is that five consecutive LR rIPC and IPC interventions increased the participants’ maximal performance, as indicated by enhanced time to exhaustion and power at exhaustion values. In contrast, this positive effect was not observed as a result of the five consecutive high reflow rIPC and IPC interventions, respectively. While the tested conditions had no direct effect on RBC deformability and pulse wave velocity, the results of the recent investigation suggest a general positive correlation of the two variables and exercise capacity.

To the best of our knowledge, only few studies have evaluated the effects of long-term (r)IPC interventions on performance in which high reflow reperfusion was employed. No previously published studies discriminated between different reflow conditions in the reperfusion phase. Overall, previous publications show heterogeneous results on the effects of (r)IPC on athletic performance [18]. While most studies revealed no or adverse effects, only few describe beneficial outcomes. Jeffries et al. [28] evaluated the effects of a seven-day IPC intervention on VO_2_max, peak power output, and time to exhaustion, employing a daily bilateral lower limb I/R maneuver of 4 × 5 min. They report no change in VO_2_max after the IPC intervention compared to a sham condition. However, the authors observed a significant increase in peak power output of 5% and time to exhaustion of 9% after the IPC intervention, supporting the results of our low reflow (r)IPC interventions in the present study showing increases in power at exhaustion of 6% during LR rIPC and of 5% during LR IPC. Additionally, Lindsey et al. [29] demonstrate that Keirin cycling athletes benefit from a seven-day IPC intervention consisting of 4 × 5 min I/R cycles since performance parameters VO_2_peak and peak power output increased compared to a sham control intervention. Our results presented herein further indicated prolonged time to exhaustion and thus increased watt power at exhaustion at the aerobic 2 mmol/L lactate threshold in the low reflow IPC setting, which might also suggest improved performance capacity by this maneuver. Such changes were not observed in either the high reflow protocols or the (r)IPC conditions. Studies dealing with the acute effects of rIPC maneuvers show that lactate production is attenuated by (r)IPC [30]. Hence, higher power values are expected at fixed lactate thresholds. The reduced lactate accumulation rate has been suggested to relate to improved muscle blood flow [31], which might promote lactate removal and transport. It is also speculated whether muscle lactate production might be reduced by IPC [32], possibly by lower energy metabolism during ischemia. It was also recently shown that a seven-day bilateral lower limb IPC protocol increases oxidative capacity and microvascular blood flow, supporting the hypothesis that especially aerobic performance might benefit from IPC maneuvers. Those studies did not report on reflow conditions and it is assumed that a high reflow reperfusion was administered [33], while the improvement described herein was only achieved in a low reflow setting. Comparative studies on the effects of different reflow conditions are lacking, and therefore, it can only be speculated what might explain the observed differences. A prolonged episode of ischemia followed by reperfusion was related to a phenomenon called ischemia reperfusion injury. This multifactorial condition includes the formation of reactive oxygen species and oxidative stress, calcium overload, and inflammation [34]. While (r)IPC has been found to reduce I/R injury [35], the exact mechanism is still debated. It might be reasonable that (r) IPC modulates the production of vasoactive substances including NO [10,35] and oxygen free radicals and reduces the overproduction of ROS [36], thus providing protection against longer periods of ischemia/reperfusion [37]. However, short I/R episodes during (r)IPC, especially those with high reflow conditions, might possibly promote an adverse environment in terms of exercise improvement. This might relate to the high shear load within the vessels and to the RBC during prompt reperfusion, which might be limited in a low reflow setting and thus which might account for the enhancements observed herein. Since this was the first report on low reflow reperfusion conditions, underlying mechanisms of the observed changes should be the focus of future research.

RBC deformability was identified as one physiological mechanism that affects aerobic performance [38,39,40,41]. During aerobic and anaerobic physical performance, such as the incremental bicycle and arm crank ergometer tests performed in this study, the smallest capillaries with a diameter smaller than the diameter of the RBC need to be passed by the RBC to cover the elevated oxygen demand of the working musculature, and high RBC deformability has been shown to be advantageous for these events [38,39,40,41]. Results of the present study indicate that RBC deformability, presented as the SS1/2:EImax ratio, remained unaffected by the tested high reflow conditions and was only affected to a minor degree by the low reflow (r)IPC maneuvers. The significant increases reported in RBC deformability were less than 4%, according to which it appears unlikely that this difference, measured in vitro, might be physiologically relevant. In contrast, a previous study by our group indicated a significant increase in RBC deformability after an acute rIPC intervention with 4 × 5 min of ischemia and high reflow [10] and related to an increase in RBC-NOS activation, which is known to affect RBC deformability. However, another investigation reported that a high reflow rIPC maneuver consisting of 4 × 5 min I/R cycles with subsequent performance testing only showed improved performance and increased RBC deformability in so-called responsive subjects, while these values were unaffected in the remaining subjects [42]. The different outcomes of the cited studies might be explained by the varying activity levels of the cohorts tested. Subjects with higher fitness levels were described to show higher deformability values because of a larger proportion of young and thus more flexible RBC [13,40]. Indeed, data of the present investigation confirm that high RBC deformability is associated with improved exercise performance. This finding suggests that physically active subjects with high aerobic capacity, as tested herein and in the study of Tomschi and colleagues [42], might require a higher (r)IPC stimulus such as the occlusion of the lower limbs to occlude more/larger tissue and vessels than untrained subjects, as previously tested [10]. This might also explain why the IPC protocol was more effective compared to the rIPC protocol. While only a comparatively small muscle mass had to be activated to measure performance using a hand crank ergometer, the performance test using a bicycle ergometer required the activation of large muscle groups. Thus, the occlusion of the small area of the upper arm might not be sufficient to generate and release adequate amounts of effector molecules to affect those large muscle groups. Thus, future studies should also test the outcome after the occlusion of either arms or thighs.

In the present study, we also investigated the effects of the different (r)IPC maneuvers on arterial stiffness-related parameters because it was shown that lower arterial stiffness, indicated by PWV, is accompanied by increased aerobic performance [43,44,45], which was confirmed by the present correlation analysis showing that lower PWV is associated with prolonged TTE. Thus, IPC improves endothelium-dependent vasodilation, and improved endothelial function might be beneficial for the regulation of vascular tone—a key component in regulating arterial stiffness and BP [46]. The results of the present study reveal that none of the employed recurrent (r)IPC maneuvers induced significant changes in PWV or blood pressure. A study by Müller and colleagues also revealed no acute effect of an IPC maneuver consisting of 3 × 5 min I/R cycles on PWV, central systolic blood pressure, peripheral blood pressure, and heart rate, using the same device as the present study [46], while a study by Grau and colleagues showed reduced peripheral systolic blood pressure immediately after rIPC, indicating an acute effect that might only last for a very short time [10]. The present data further suggest that recurring (r)IPC cycles did not influence PWV or central and peripheral blood pressure. However, it was shown that repeated IPC stimuli augment endothelium-dependent vasodilation in humans through increases in NO and endothelial progenitor cell production [47]. Most likely, these arterial stiffness lowering effects might be particularly effective in humans with abnormal arterial compliance and, hence, elevated blood pressure and high arterial stiffness. Consequently, repeated, multi-day (r)IPC interventions seem to be particularly effective in clinical settings [48]. Due to the fact that the participants of the present study exhibited blood pressure and PWV values in a normal range, significant changes in these parameters were improbable.

## 5. Conclusions

The results of the present study indicate, for the first time, that both a five-day low reflow rIPC and a five-day low reflow IPC intervention increased peak power in the respective performance tests, which was not observed in the high reflow interventions. Hence, more attention should be paid to the reperfusion phase of (r)IPC protocols as the shear stress during reperfusion might be a crucial factor for inducing performance-enhancing effects. The observations were only related to changes in RBC deformability to a lesser extent, although RBC function affects the performance outcome. Additionally, blood pressure and pulse wave velocity were not altered during (r)IPC, assuming that RBC deformability and arterial stiffness affect exercise performance, but in contrast were only affected by the (r)IPC stimuli tested herein to a lesser extent. The knowledge gained by the present study might be of interest for sport scientists, coaches, and athletes who seek to improve training and/or competition performance by the means of (r)IPC applications, as for the first time this study brings forward the theory that performance might be enhanced when the reflow velocity is intentionally reduced. On the other hand, the obtained results can be seen and utilized as a basis for future research to investigate mechanisms occurring in response to low reflow (r)IPC interventions.

## Figures and Tables

**Table 1:** Resting peripheral and central blood pressures (BP) for the different rIPC and IPC protocols. Data are shown as mean (SD).

Peripheral BP		Pre		Post	
		RR Sys (mmHg)	RR Dias (mmHg)	RR Sys (mmHg)	RR Dias (mmHg)
**IPC**	Low Reflow	127.3 (9.5)	70.7 (6.3)	130.0 (14.8)	71.6 (9.7)
	High Reflow	123.1 (9.5)	68.6 (7.2)	121.0 (9.5)	69.1 (6.2)
**rIPC**	Low Reflow	125.0 (7.1)	69.7 (7.6)	125.7 (7.6)	68.6 (8.7)
	High Reflow	126.0 (12.7)	69.0 (7.8)	127.3 (11.9)	70.6 (7.7)
**Central BP**		**Pre**		**Post**	
		RR Sys (mmHg)	RR Dias (mmHg)	RR Sys (mmHg)	RR Dias (mmHg)
**IPC**	Low Reflow	119.7 (10.9)	71.9 (6.8)	118.8 (13.1)	70.3 (7.5)
	High Reflow	114.2 (11.5)	70.0 (6.2)	112.0 (13.3)	70.4 (7.0)
**rIPC**	Low Reflow	116.7 (9.3)	70.3 (7.8)	113.6 (11.3)	70.3 (7.7)
	High Reflow	112.7 (11.5)	71.0 (8.4)	113.3 (9.9)	73.0 (8.9)

## Data Availability

The data that support the findings of this study are available upon reasonable request from the corresponding author. The data are not publicly available due to privacy or ethical restrictions.
